# Correlation Between Radiological and Functional Outcomes Following Operative and Nonoperative Management of Acetabular Fractures: A Prospective Observational Study

**DOI:** 10.7759/cureus.58146

**Published:** 2024-04-12

**Authors:** Harshvardhan Buddhist, Shivam Sinha, Ravikant Maurya, Muhammad Wamique Ansari, Krishan Kumar, Amrit Panda, Soumya Ranjan Nayak, Rahul Rai, Vikas Rao, Sushil Gandhi Poddar

**Affiliations:** 1 Orthopaedics, Institute of Medical Sciences, Banaras Hindu University, Varanasi, IND; 2 Orthopaedics and Traumatology, Institute of Medical Sciences, Banaras Hindu University, Varanasi, IND; 3 Orthopaedics, Sardar Patel Medical College, Bikaner, Delhi, IND; 4 Orthopaedics, SCB (Srirama Chandra Bhanja) Medical College and Hospital, Cuttack, IND; 5 Orthopaedics, Vardhman Mahavir Medical College and Safdarjung Hospital, Delhi, IND

**Keywords:** radiographic outcome, function, conservative, surgery, acetabular fracture

## Abstract

Introduction: The management of acetabular fractures is a complicated orthopedic procedure that has been advancing with time. Newer radiological tools like CT scans help surgeons to identify and manage these fractures more attentively. The study was conducted to evaluate the clinical and radiographic outcomes in patients with acetabular fractures managed either conservatively or by open reduction and internal fixation.

Materials and method: The study was done on 35 patients aged 18-60 years, with acetabular fractures treated either surgically or conservatively. Clinical scorings and radiological scoring were only taken and noted at three- and six-month intervals using Matta’s radiographic scoring and modified Merle d’Aubigne and Postel clinical hip scoring. Clinico-radiological variables and complications were compared between the two groups. The data obtained was subjected to statistical analyses using IBM Statistical Package of Social Sciences (SPSS) 2.0 version software (Chicago, IL, USA) at a level of significance being p<0.05.

Results: Out of a total of 35 patients, 19 were treated surgically and 16 conservatively. In patients belonging to the surgical treatment group, a maximum of 57.9% were aged 40-50 years, whereas the maximum patients (50%) of the conservative treatment group were aged <40 years, with male predominance in both groups. The type of fracture was recorded according to Judet and Letournel in both groups. Merle d'Aubigne's scoring and Matta's hip score were recorded at three and six months in both groups. A positive correlation was seen between radiological and functional outcomes at three and six months, which means that the higher the radiological scoring, the better the functional outcome of the patient managed either conservatively or surgically in the entire cohort.

Conclusion: Our study revealed that surgically managed patients had better functional and radiological outcomes than the patients who were conservatively managed at six months of follow-up. However, this is associated with more complications depending on fracture complexity and initial presentation of hip dislocation. The higher the radiological scoring, the better the functional outcome of the patient managed either conservatively or surgically in the entire cohort.

## Introduction

Acetabular fractures represent a significant subset of orthopedic injuries and often occur concomitantly with other potentially life-threatening conditions such as pelvic traumas and hip dislocations. They account for approximately 10% of all pelvic injuries, with the majority attributed to motor vehicle accidents (over 80%) and falls from significant heights (10.7%) [1]. Managing acetabular fractures poses a considerable challenge for orthopedic surgeons due to their complex nature and the potential for devastating consequences if left untreated [2]. Historically, conservative management was attempted for acetabular fractures, particularly in cases of non-displaced fractures. However, this approach yielded suboptimal outcomes, especially in instances of fracture displacement. One of the primary limitations was the inability to accurately evaluate the fracture in three dimensions prior to surgical intervention. The advent of modern imaging modalities, such as computed tomography (CT) scans, has revolutionized the diagnosis and treatment planning for acetabular fractures, enabling surgeons to precisely assess fracture patterns and plan appropriate interventions [3]. Despite technological advancements, surgical management of acetabular fractures remains intricate and demanding, particularly for trauma surgeons. While some types of acetabular fractures may not necessitate surgical intervention, fractures causing hip instability, incongruity, or displacement in the superior acetabular region often require internal fixation or open reduction to achieve satisfactory outcomes. CT imaging, coupled with three-dimensional reconstruction, has emerged as a valuable tool in the management of acetabular fractures, facilitating enhanced visualization and planning for surgeons, including those who may be less experienced or unfamiliar with these complex injuries [4]. However, challenges persist in the timely evaluation and treatment of acetabular fractures, stemming from delays in radiological assessment and variations in surgical expertise. To address these challenges and evaluate the clinical and radiological outcomes of acetabular fracture management, a prospective comparison study was initiated at our institution. This study aims to assess the efficacy of conservative versus surgical approaches, specifically open reduction and internal fixation, in patients presenting with acetabular fractures. By systematically analyzing patient outcomes and radiological findings, we seek to contribute valuable insights into the optimal management strategies for acetabular fractures and improve patient care in this challenging clinical scenario.

## Materials and methods

The present prospective, non-randomized study was conducted at the Institute of Medical Sciences, Banaras Hindu University, Varanasi, Uttar Pradesh, India, after obtaining approval from the institutional ethics committee (IRB no. 2874) approved by Dr. Kiran Giri on August 31, 2021. A prospective study design was chosen as it is considered the gold standard for evaluating outcomes and establishing causal relationships. The study included 35 patients aged between 18 and 60 years with closed acetabular fractures. The age criteria were set based on previous studies which have shown that acetabular fractures predominantly occur in young to middle-aged adults, with a peak incidence in the fourth decade of life. Both male and female patients who were skeletally mature, provided written informed consent, and were willing to come for regular follow-up visits were included. Skeletally mature patients were chosen to avoid complications related to growth plate injuries. Obtaining informed consent is an ethical requirement for research involving human participants. Patients with open fractures, existing limb deformities, medical comorbidities, pathological fractures, and other injuries in the ipsilateral limb were excluded to eliminate confounding factors that could affect treatment and outcomes. After initial resuscitation and splintage, patients underwent a detailed clinical examination and radiographic evaluation as per standard protocols. Plain radiographs, comprising an anteroposterior view of the pelvis along with Judet oblique views, alongside computed tomography scans, are deemed indispensable for preoperative planning in cases of acetabular fractures. The fractures were classified according to the widely used Judet and Letournel system based on the CT scan findings. Patients were counseled regarding the treatment options of conservative management versus surgical intervention with open reduction and internal fixation. Those unwilling for surgery or deemed unfit due to medical reasons were managed conservatively with skeletal traction and mobilization as tolerated, as per established protocols. For surgical management, the Kocher-Langenbeck approach was used in most cases, which is a standard approach for acetabular fracture fixation providing wide exposure. Surgeries were performed by experienced trauma surgeons at the institute. Postoperatively, patients were started on antithrombotic prophylaxis and rehabilitation protocols as per institutional standards, which are crucial for preventing complications and optimizing functional outcomes. Follow-up evaluations were carried out at one, three, and six months, which is a commonly followed schedule for assessing outcomes in acetabular fracture studies. At the three- and six-month follow-ups, clinical assessment was done using the modified Merle d'Aubigne and Postel hip scoring system, which is a validated instrument for evaluating hip function. Radiological evaluation was performed using Matta's criteria, which is a reliable method for assessing the quality of reduction and congruity of the acetabular joint surface. Complications and reoperations, if any, were also noted. The data was compiled and analyzed using IBM Statistical Package of Social Sciences (SPSS) Statistics 2.0 version software (Chicago, IL, USA), which is a widely used statistical package in biomedical research. Appropriate statistical tests were applied based on the nature and distribution of the data. A p-value less than 0.05 was considered statistically significant, which is a standard threshold in most studies.

## Results

Out of a total of 35 patients, 19 were treated surgically and 16 conservatively. In patients belonging to the surgical treatment group, a maximum of 57.9% were aged 40-50 years, whereas the maximum patients (50%) of the conservative treatment group were aged <40 years, with male predominance in both groups. Chi-square statistical analysis revealed an insignificant difference (p-value >0.05) among both groups in relation to age and gender. We also found that in both groups, the maximum number of patients was injured due to road traffic accidents, followed by falls from height, with an insignificant difference (p-value >0.05) between both groups statistically in relation to the mechanism of injury. Bicolumnar fractures were the most common pattern in the surgical group, whereas posterior column fractures were the most common fractures in the conservative groups (Table 1).

**Table 1:** Type of fracture according to Judet and Letournel

Type of fracture	Surgical treatment		Conservative treatment		Total
	Frequency	Percentage	Frequency	Percentage	
Anterior column	0	0.0%	2	12.5%	2
Anterior column+posterior hemitransverse	0	0.0%	2	12.5%	2
Bicolumnar	9	47.4%	2	12.5%	11
Bicolumnar with iliac wing	1	5.3%	0	0.0%	1
Posterior column	4	21.1%	5	31.3%	9
Posterior wall	4	21.1%	3	18.8%	7
Posterior wall+anterior wall	0	0.0%	1	6.3%	1
T type fracture	0	0.0%	1	6.3%	1
Transverse	1	5.3%	0	0.0%	1
Total	19	100.0%	16	100.0%	35

At six months, the surgically treated group had significantly better Merle d'Aubigne scores compared to the conservative group (Table 2).

**Table 2:** Merle d'Aubigne scoring at 3 and 6 months *p-value <0.05 is significant.

Merle d'Aubigne scoring at 3 months	At 3 months				Merle d'Aubigne scoring at 6 months	At 6 months			
	Surgical treatment		Conservative treatment			Surgical treatment		Conservative treatment	
	N	%	N	%		N	%	N	%
5	0	0.0%	1	6.3%	11	3	15.8%	2	12.5%
7	1	5.3%	0	0.0%	12	1	5.3%	6	37.5%
8	1	5.3%	2	12.5%	13	3	15.8%	5	31.3%
9	5	26.3%	8	50.0%	14	5	26.3%	1	6.3%
10	7	36.8%	4	25.0%	15	5	26.3%	1	6.3%
11	2	10.5%	1	6.3%	16	2	10.5%	0	0.0%
12	3	15.8%	0	0.0%					
Chi square	6.971				11.29				
p-value	0.324				0.046*				

The Matta hip scores, assessing the radiological outcome at three and six months, are shown in Table 3. There was no significant difference in the Matta scores between the two groups at either time point.

**Table 3:** Matta hip score at 3 and 6 months *p-value >0.05 is insignificant.

Matta hip score at 3 months	At 3 months				At 6 months				
	Surgical treatment		Conservative treatment		Surgical treatment		Conservative treatment		
	N	%	N	%	N	%	N	%	
Excellent	1	5.3%	0	0.0%	1	5.3%	0	0.0%	
Fair	5	26.3%	11	68.8%	5	26.3%	9	60.0%	
Good	9	47.4%	3	18.8%	11	57.9%	5	33.3%	
Poor	4	21.1%	2	12.5%	2	10.5%	1	6.7%	
Chi square	6.709				5.51				
p-value*	0.082				0.239				

Complications observed during the study period in each group are tabulated in Table 4. The most common complication was avascular necrosis (AVN) hip in surgically treated patients. Complications were fewer in the conservative group as compared to the surgical groups.

**Table 4:** Complications AVN: avascular necrosis.

Complications	Surgical treatment		Conservative treatment		Total
	Frequency	Percentage	Frequency	Percentage	
No	14	73.7%	14	87.5%	2
AVN hip	3	15.8%	1	6.3%	2
Femur head collapse	1	5.3%	0	0.0%	11
Myositis ossificans	1	5.3%	0	0.0%	1
Neck of femur fracture	0	0.0%	1	6.3%	9
Total	19	100.0%	16	100.0%	35

The correlation between radiological (Matta) and functional (Merle d'Aubigne) outcomes at three and six months, analyzed using Spearman's Rho, is depicted in Table 5. For the entire cohort, there was a positive and significant correlation between the two scoring systems at both follow-up times. However, the correlation varied between the surgical and conservative groups over time.

**Table 5:** Correlation between radiological and functional outcomes at 3 and 6 months p-value <0.05 is significant.

Spearman's rho		Matta at 3 months	Matta at 6 months
Merle (Conservative treatment)	Correlation coefficient	0.422	-0.159
	p-value	0.104	0.572
	N	16	15
Merle (Surgical treatment)	Correlation coefficient	0.420	0.626
	p-value	0.073	0.004*
	N	19	19
Merle (Whole sample)	Correlation coefficient	0.472	0.444
	p-value	0.004*	0.008*
	N	35	34

## Discussion

Surgery was used to maintain the articular surface, especially in the weight-bearing area, by obtaining a suitable exposure and anatomical reduction. In contrast, whether the patient is not healthy enough for surgery or is unwilling to have surgery, conservative therapy aids in sustaining the articular reduction. About the superiority of one modality over another, there is still a dilemma. Our study aims to contrast these two treatment approaches and determine whether certain aspects of articular reduction are correlated with functional outcomes.

In a study by Wang et al., selected acetabular fractures were included on the basis of Letournel criteria, and patients were followed up for 3.5 years [5]. Unlike our study, where we included all fractures being classified under Judet and Letournel classification, with the maximum being bicolumnar fractures. This may be due to more number of younger patients with high-speed traumas, who reported to our tertiary care center. Nevertheless, our study was followed for a short term with the maximum follow-up of some of the patients only for two years. For establishing the correlation of clinical-radiological outcome with the two modalities, we compared the Merle scoring at three months between the groups. We found that three patients who were surgically managed had the best clinical outcome at the end of three months of follow-up. At six months, two patients who were surgically managed had the best clinical scoring outcomes and better scores than the patients managed conservatively. In a study by Lovrić et al., it was observed that anterior column fractures that were fixed surgically healed three times faster and revealed a better functional outcome as compared to those patients conservatively managed patients [6]. In contrast, in patients with transverse fractures, the functional status was better and the treatment period was shorter than after the conservative treatment. Similarly, in our study, we tried to manage anterior column and posterior wall fractures conservatively, whereas bicolumnar fractures were treated surgically. Lovrić et al. observed better outcomes among surgically managed patients for every fracture [6]. In contrast, our study revealed good results even in anterior column fractures that were managed conservatively. Being a smaller cohort and with different demographics, the data of our study regarding a particular fracture type may not be generalized but provides a new insight into the dilemma of acetabular fracture. Magu et al. observed that patients with congruent reduction had good or excellent functional outcomes [7]. Good to excellent radiologic and functional outcomes were achieved in patients with posterior wall fractures. Nonoperative treatment of acetabular fractures gives good radiological and functional outcomes if there is a congruent reduction. Similarly in our study, we found that patients managed surgically and with congruent reduction had a better functional outcome than those who had an incongruent reduction.

In our study, we found a positive correlation between radiological and functional outcomes, revealing that with a good reduction radiologically, patients also had a good functional outcome for all types of acetabular fractures. Our study showed that at the end of three and six months, surgically treated patients had a better radiological score than the conservatively managed patients. Some patients had a complication during the study period.

At the end of three and six months, we found a positive and significant correlation between functional and radiological aspects. This indicated that as the radiological scoring increased, the functional score also increased for the entire cohort. For the conservative group, a positive and insignificant correlation was observed between functional and radiographical outcomes at three months, but at six months, we found a negative and insignificant correlation between both outcomes. In the surgically managed group, a positive and insignificant correlation was observed between functional and radiographical outcomes at three months. However, at the end of six months, there was a positive and significant correlation between both outcomes.

Alpaydın et al. observed that there was a statistically insignificant difference in functional scores between both groups (p>0.05) [8]. Matta’s radiological staging score was significantly higher in the operated group, which was not directly correlated with functional outcome. Similarly in our study, surgically managed patients had a better functional score at six months than conservatively managed patients. However, a positive and significant correlation was observed between the functional and radiological scores at three and six months. Mohan et al. recommended conservative management in the elderly on the basis of injury and surgical factors [9]. They had advised a multidisciplinary approach focused on early mobility, with minimization of risk and regular follow-up to optimize the outcomes of the patients. This is somewhat similar to our study as we also managed patients conservatively who were elderly and had any other risk factors for surgery. The outcome was also acceptable. Grubor et al. observed that functional outcomes were good in the maximum number of patients (56%) [10]. They concluded that conservative management is acceptable if the fracture displacement is less than 5 mm. Surgical treatment was advised in patients with incongruent or unstable fractures having verified displacement of more than 5 mm. Similarly, we also managed patients surgically, who had incongruent hip and associated displacement not manageable with traction. But our results did not coincide with every patient as there were outliers as well. Two patients had a Matta score of 14 at the end of six months, but they had a fair Merle scoring, revealing that despite having a good clinical function these patients had a fair radiological function. One patient who had an excellent Matta score at the end of six months had a functional score of only 12 at the end of six months, contradicting the fact that we had a positive correlation between the radiological and functional scores at the end of our study, thus proving that the correlation exists, but it is not very strong. One patient who was surgically managed had severe myositis ossificans of the hip region at the end of six months and stiffness of the hip joint.

Strength of study

The study is based on comparable groups leading to a better study design. It follows standard classification systems for both functional and radiological comparison of acetabular fractures. There were many studies on the outcomes of surgical treatment with fewer studies focusing on conservative management. Our study compared both management techniques using radiological and functional scorings. Moreover, the serial correlation of acetabular fracture management added a new dimension to the understanding of outcomes and reduction of fracture in either modality of treatment.

Weakness of study

The sample size is less due to low patient inflow during the COVID-19 pandemic. Many patients were lost to follow-up due to COVID pandemic and unwillingness to attend outpatient department (OPD) physically. The study includes all types of acetabular fractures and is not confined to any single fracture pattern. Less follow-up time was available.

## Conclusions

Our study revealed that surgically managed patients had better functional and radiological outcomes than the patients who were conservatively managed at six months of follow-up. However, this is associated with more complications depending on fracture complexity and the initial presentation of dislocation of the hip. In the conservative group at six months, a negative but insignificant correlation was seen between the scores, which implies that even if the radiological outcome was not good, the functional outcome was acceptable. A positive correlation was seen between radiological and functional outcomes at three and six months, which means that the higher the radiological scoring, the better the functional outcome of the patient managed either conservatively or surgically in the entire cohort.

## References

[1] Giannoudis PV, Grotz MR, Papakostidis C, Dinopoulos H (2005). Operative treatment of displaced fractures of the acetabulum: a meta-analysis. J Bone Joint Surg Br.

[2] Kebaish AS, Roy A, Rennie W (1991). Displaced acetabular fractures: long-term follow-up. Trauma.

[3] Anizar-Faizi A, Hisam A, Sudhagar KP, Moganadass M, Suresh C (2014). Outcome of surgical treatment for displaced acetabular fractures. Malays Orthop J.

[4] Durkee NJ, Jacobson J, Jamadar D, Karunakar MA, Morag Y, Hayes C (2006). Classification of common acetabular fractures: radiographic and CT appearances. AJR Am J Roentgenol.

[5] Wang P, Zhu X, Xu P, Zhang Y, Wang L, Liu X, Mu W (2016). Modified ilioinguinal approach in combined surgical exposures for displaced acetabular fractures involving two columns. Springerplus.

[6] Lovrić I, Jovanović S, Leksan I, Biuk E, Kristek J, Radić R (2007). Functional status of hip joint after surgical and conservative treatment of acetabular fracture. Coll Antropol.

[7] Magu NK, Rohilla R, Arora S (2012). Conservatively treated acetabular fractures: a retrospective analysis. Indian J Orthop.

[8] Alpaydın E, Erem M, Çopuroğlu C (2022). Comparison of the functional and radiological results of the conservatively and surgically treated displaced acetabulum fractured patients. Ulus Travma Acil Cerrahi Derg.

[9] Mohan K, Broderick JM, Raza H, O'Daly B, Leonard M (2022). Acetabular fractures in the elderly: modern challenges and the role of conservative management. Ir J Med Sci.

[10] Grubor P, Krupic F, Biscevic M, Grubor M (2015). Controversies in treatment of acetabular fracture. Med Arch.

